# Translocation of TRPV2 channel induced by focal administration of mechanical stress

**DOI:** 10.14814/phy2.12296

**Published:** 2015-02-13

**Authors:** Masahiro Nagasawa, Itaru Kojima

**Affiliations:** Institute for Molecular & Cellular Regulation, Gunma UniversityMaebashi, Japan

**Keywords:** Calcium, mechanosensitive channel, TRP channel, TRPV2

## Abstract

The effect of focal mechanical stress on the localization of TRPV2 was investigated in HT1080 cells, where only mRNA for TRPV2 was detected among members of the TRPV channel family. Mechanical stress was applied by adding negative pressure using a glass pipette. When focal mechanical stress was applied, subplasma membrane Ca^2+^ concentration ([Ca^2+^]_s_) was increased beneath the pipette, which propagated throughout the cell. The increase in [Ca^2+^]_s_ was blocked by ruthenium red or by knocking down TRPV2. Elevation of [Ca^2+^]_s_ was not observed by removal of extracellular Ca^2+^, by an addition of a phosphatidylinositol 3-kinase inhibitor LY29034, and by transfection of dominant-negative Rac. In cells expressing GFP-TRPV2 and RFP-Akt, administration of focal mechanical stress induced accumulation of GFP-TRPV2 beneath the pipette. RFP-Akt was also accumulated to the same site. Gadolinium blocked the elevation of [Ca^2+^]_s_ induced by focal mechanical stress and also attenuated accumulation of TRPV2. When GFP-TRPV1, GFP-TRPV3, GFP-TRPV4, GFP-TRPV5, or GFP-TRPV6 was transfected ectopically in HT1080 cells, only GFP-TRPV4 was accumulated beneath the pipette in response to the focal mechanical stress. These results indicate that TRPV2 translocates to the site receiving a focal mechanical stress and increases [Ca^2+^]_s_.

## Introduction

Like other members of the transient receptor potential vanilloid channel (TRPV) family, TRPV2 is activated by various stimuli (Caterina et al. 1999; Kanzaki et al. 1999; Boels et al. 2001; Nagasawa et al. 2007; Hisanaga et al. 2009; Monet et al. 2009; Nagasawa and Kojima 2012). Caterina et al. (1999) showed that TRPV2 is activated by high temperature above 52°C. The threshold of the temperature activating the channel is the highest among the temperature-activated thermo-TRPs. Because of the high threshold temperature, the physiological role of TRPV2 in vivo as a thermo-TRP is not totally certain yet. TRPV2 is also activated by various ligands including growth factors such as insulin-like growth factors, epidermal growth factor, and platelet-derived growth factor (Kanzaki et al. 1999). It is also activated by insulin and other cytokines (Kanzaki et al. 1999; Boels et al. 2001; Nagasawa et al. 2007; Hisanaga et al. 2009; Monet et al. 2009). These TRPV2-activating ligands all activate phosphatidylinositol (PI) 3-kinase and downstream signaling molecules including a small G-protein Rac (Kanzaki et al. 1999; Boels et al. 2001; Nagasawa et al. 2007; Hisanaga et al. 2009; Monet et al. 2009; Nagasawa and Kojima 2012). In fact, these ligands activate PI 3-kinase and Rac and induce translocation of TRPV2 from an intracellular pool to the plasma membrane (Boels et al. 2001; Nagasawa et al. 2007; Hisanaga et al. 2009; Nagasawa and Kojima 2012). It appears that these ligands regulate TRPV2 mainly by inducing translocation of TRPV2. However, it is not certain whether or not these ligands also act on TRPV2 and modulate gating of the channel.

TRPV2 was also shown to be activated by membrane stretch and has been thought to be a mechanosensitive channel (Iwata et al. 2003; Muraki et al. 2003). In vascular smooth muscle cells, exposure to hypotonic solution leads to activation of a nonselective cation channel current, which results in an elevation of cytoplasmic free Ca^2+^ concentration ([Ca^2+^]_c_). These responses are blocked by ruthenium red, an inhibitor of the TRPV channels, and by knockdown of TRPV2 (Muraki et al. 2003). Therefore, TRPV2 may act as a stretch-activated calcium-permeable channel in vascular smooth muscle cells. TRPV2 is also expressed in cardiomyocytes, and membrane stretch activates TRPV2 in these cells (Iwata et al. 2003). Interestingly, Iwata et al. (2003) reported that membrane-stretch induced translocation of TRPV2 to the plasma membrane. This raises the possibility that trafficking of TRPV2 is regulated by the membrane stretch. If TRPV2 is constitutively active, in other words if opening and closure of the channel occur spontaneously, translocation of TRPV2 to the plasma membrane would facilitate calcium entry. In a strict sense, however, it is not clear at present whether or not membrane stretch modifies the gating of TRPV2.

In this study, we investigated the activation mechanism of TRPV2 in response to mechanical stress. Specifically, we examined whether or not focal administration of mechanical force induced translocation of TRPV2 to the site receiving the mechanical stress. We also addressed whether membrane stretch directly activated TRPV2 by modifying the gating.

## Materials and Methods

### Cell cultures

HT1080, a human fibrosarcoma cell line, was purchased from ATCC (Manassas, VA). The cells were cultured in α-modified Eagles medium (α-MEM) (Invitrogen, Carlsbad, CA) supplemented with 10% fetal bovine serum (FBS). For production of adenovirus, HEK 293 cells were grown in Dulbecco's modified Eagle's medium (DMEM) supplemented with 10% FBS and antibiotics. The transient transfection was carried out using lipofectamine 2000 (Invitrogen).

### Adenovirus constructs

TRPV2 with an exofacial Myc epitope and EGFP, Strawberry or KATE (hTRPV2-Myc-EGFP, -Strawberry, -KATE) and PH mAkt-TagRFP adenoviral constructs were described previously (Nagasawa and Kojima 2012). Similarly, the short harpin RNA (shRNA) vector approach was used to knockdown human TRPV2 mRNA. We previously reported the knockdown constructs for mouse TRPV2 mRNA (Nagasawa and Kojima 2012). Human knockdown vectors for TRPV2 were similarly prepared. Briefly, the promoter of mU6sh-EGFP/RFP-pENTR vectors was replaced by the hU6 promoter (hU6shEGFP/RFP-pENTR). The human U6 (hU6) promoter was derived from the pENTR/U6 vector (Invitrogen). The shRNA target sequence of two complementary single-strand oligos GATCCGTTGGGGATCGTTGCCTTTCAGTGTGCTGTCCTGAAAGGCAATGATCTCCAGCTTTTTTAT and CGATAAAAAAGCTGGAGATCATTGCCTTTCAGGACAGCACACTGAAAGGCAACGATCCCCAACG were synthesized (Invitrogen). The two oligos were annealed and cloned into the BamH1 and Cla1 sites of hU6sh EGFP-pENTR and hU6sh RFP-pENTR vectors, respectively. The stuffer sequence derived from hU6sh EGFP or RFP was used as a negative control. Yellow Cameleon 3.6 pm (YC-pm) was a kind gift from Dr. Atsushi Miyawaki (Riken, Wako, Japan). Yellow Cameleon-nano plasmid (Horikawa et al. 2010) was a kind gift from Dr. T Nagai (Osaka,University, Osaka, Japan). Modified-Yellow Cameleon-nano-pm (pm-Cameleon-nano) was used in the experiments. Briefly, a sequence between ClaI and Bgl II restriction enzyme sites of YC-pm was replaced with Yellow Cameleon-nano 15, and ECFP of Yellow Cameleon-nano15 was exchanged by mTFP. mTFP was purchased from Allele Biotechnology (San Diego, CA). The dominantly negative rat dynamin II mutant was a kind gift from Kazuo Kasai (Tsukuba University, Tsukuba, Japan). Strawberry was fused to the N-terminal of rat dynamin II and subcloned into Gateway entry vector, pENTR3C (Invitrogen). Adenovirus vectors were made by using the Gateway technology (Invitrogen).

### Real-time PCR

cDNA was synthesized using the Superscript III First-Strand Synthesis System (Invitrogen) and primed with human actin or human TRPV2 gene-specific primers. The synthesized cDNA was added to the 2 × SYBR Green PCR Master Mix (Applied Biosystems, Carlsbad, CA) in the presence of 5′ and 3′ gene specific primers according to the manufacturer's instructions. The quantitative analysis was performed by the 7500 Fast Real-Time PCR system (Applied Biosystems). Real-time PCR primers and actin GSP primer are shown as follows in Table1.

**Table 1:** Primers used in RT-PCR and real-time PCR

	Sense primers	Antisense primers	GSP prime
hTRPV1	5′-CCACAGAGGATCCAGCAAGGATGAAGAAATGGA	5′-TTGACAGTGCTGTCTGCGTGACGTCCTCA	5′-TGCTGACAGAGCACTGGTGTTC
hTRPV2	5′-TCCTAGGATGACCTCACCCT	5′-GCCATCAGTTGGACTGGAG	5′- GCCAGCAGATGTGGTTGGAAAG
hTRPV3	5′-CAGAACCTCACCAGCCATGAAAGCCCAC	5′-AGCTCTGGGTTCCGCTTCTACACCGAG	5′-TCAAAGCCTCTCTGCACAGAGTC
hTRPV4	5′-ATTCAGGAAGCGCGGATCTCCCG	5′-GTCCCTAGAGCGGGGCGTCATCA	5′-TAGAAATGAGTGGGCAGAGAA
hTRPV5	5′-ATTCTATAATCTGCCAGTGTCTGCAAGGA	5′-ATAGCGATGTTAATCAAAAATGGTAGACCTCC	5′-CCAACCGGGAGTAAGGTCAAGA
hTRPV6 (real time PCR)	5′-TACACCCCATGGGTTTGTCACTG	5′-AACACGCAGTCAGATCTGATATTCCCA	5′-ACCCAGGAAAATGAGAGCAAGT
hTRPV2	5′-GGAGGTGAACTGGGCTTCATG	5′-GCACCATCCTCATCCTCCTTG	
Actin	5′-GAGGCACTCTTCCAGCCTTC	5′-CGGATGTCCACGTCACACTTC	5′-GTAACGCAACTAAGTCATAG

### Imaging experiments

Epifluorescence microscopy images and FRET images were captured using an inverted microscope (IX-81, Olympus, Tokyo, Japan) equipped with a cooled 3CCD camera (AQUACOSMOS/ASHURA, Hamamatsu Photonics, Hamamatsu, Japan). The cells expressing respective constructs were incubated in Hank's balanced salt solution containing 1.3 mmol/L Ca^2+^ (Nissui, Tokyo, Japan). The cells were incubated at 30–35°C by a heated plate (Thermoplate, Olympus). Live cell images were acquired at 15 sec intervals and mechanical stimulation by a point source of negative pressure from a glass-micropipette was performed. Statistical analyses were carried out by using the Mann–Whitney's *U*-test. For immunofluorescence microscope analyses, cells were grown on glass coverslips. Cells were fixed for 30 min at room temperature in 4% paraformaldehyde PBS solution and then permeabilized for 5 min in 0.1% Triton X-100. For staining of extracellular myc-tagged hTRPV2, cells were not permeabilized. Blocking of nonspecific binding was performed by incubating cells with Block Ace (Snow Brand, Tokyo, Japan). Cells expressing myc epitope-tagged proteins were immunostained with myc 9E10 (UBI) mouse mAbs, followed by incubation with Alexa-Fluor-conjugated secondary goat anti-mouse antibodies (Molecular Probes). Images were collected by an epifluorescent microscope (IX70; Olympus) with a cooled CCD digital camera (Retiga) (QImaging, Surrey, Canada) using Qcapture Pro software (Qimaging) (Nagasawa and Kojima 2012).

### Preparation of hTRPV1-EGFP, hTRPV3-EGFP, hTRPV4-EGFP, mTRPV5-EGFP, and mTRPV6-EGFP

mRNA for hTRPV1, hTRPV3, and hTRPV4 were obtained from various cell lines as mentioned above. mTRPV6 was obtained from mouse placenta. mTRPV5 plasmid was purchased from Thermo Fisher Scientific (Pittsburgh, PA). EGFP was fused in the C-terminal in frame and pENTR vectors were made. Adenovirus constructs were prepared as mentioned above.

## Results

**Figure 1:**
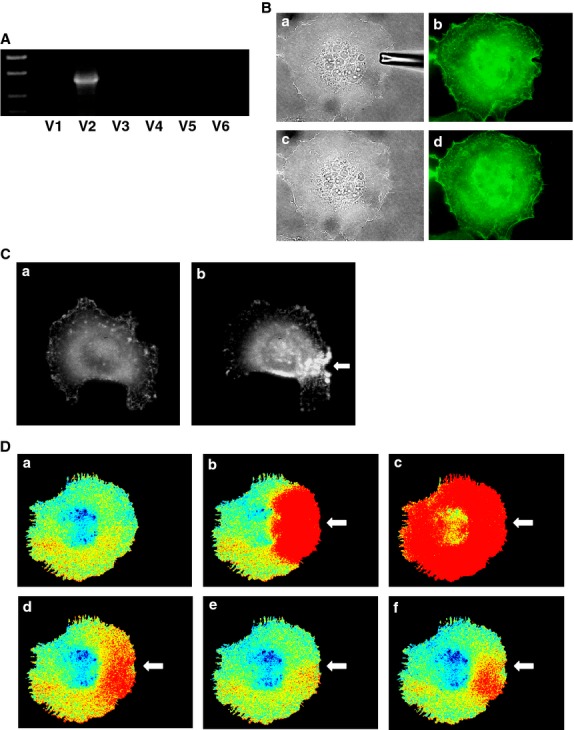
Effect of focal application of mechanical stress on [Ca^2+^]_s_. (A) Expression of members of the TRPV family in HT1080 cells. mRNA expression of the members of the TRPV2 family was measured by RT-PCR. (B) Reversibility of the plasma membrane. Mechanical stress was applied using a glass pipette (a, b). The plasma membrane recovered 1 min after the removal of the pipette (c, d). a, c: bright field, b, d: plasma membrane was made visible by transfection of PM-GFP. (C) Effect of focal application of mechanical stress on the localization of Zyxin. Focal mechanical stress was applied to the cell for 5 min as indicated by the arrow (b). Localization of zyxin was determined by monitoring fluorescence of GFP-zyxin. a: before application of the mechanical stress. (D) Changes in [Ca^2+^]_s_ in a Cell Receiving Focal Mechanical Stress. Focal mechanical stress was applied as indicated by the arrow and changes in [Ca^2+^]_s_ were monitored using pm-Cameleon-nano. Warm color indicates elevation of [Ca^2+^]_s_. The results are representative of those obtained in five experiments. a: 0 min, b: 0.5 min, c: 1 min, d: 2 min, e: 5 min, f: 10 min.

**Figure 2:**
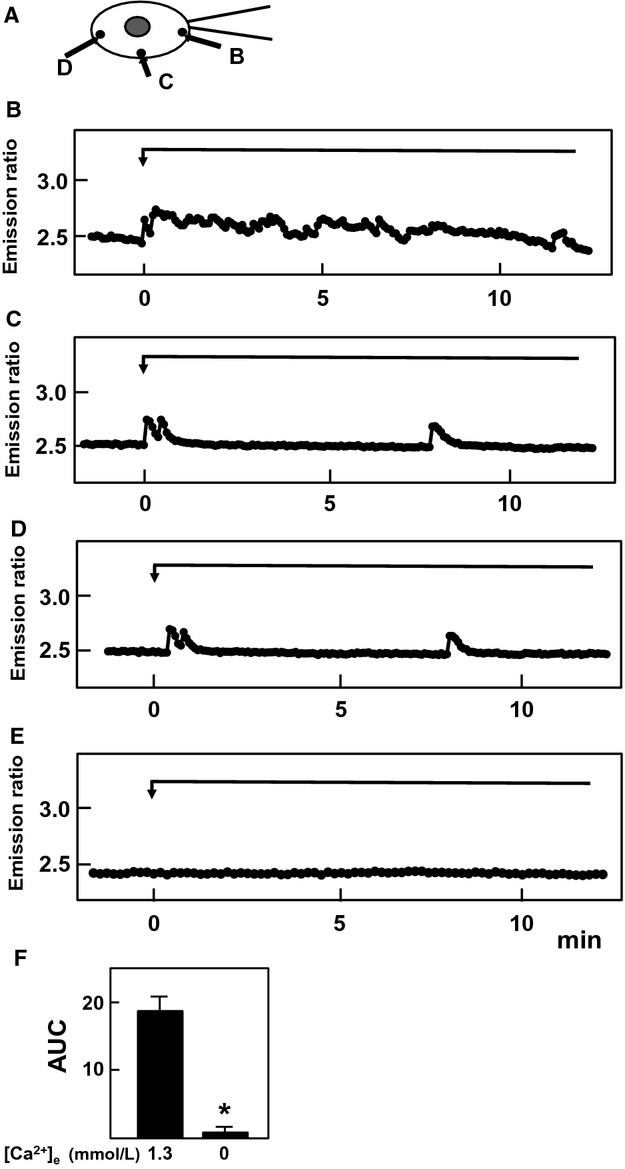
Effect of focal administration of mechanical stress on [Ca^2+^]_s_. Focal mechanical stress was administered using a pipette (A) and changes in [Ca^2+^]_s_ at B, C and D were monitored by using pm-Cameleon-nano. E: Changes in [Ca^2+^]_s_ at B in the absence of extracellular calcium. Results are representative of five experiments. (F) Effect of removal of extracellular calcium on elevation of [Ca^2+^]_s_. Experiments were carried out as shown in Fig.2B and 2E and area under the curve (AUC) was calculated. Values are the mean ± SE for five experiments. *P <* *0.01 versus 1.3 mmol/L calcium.

**Figure 3:**
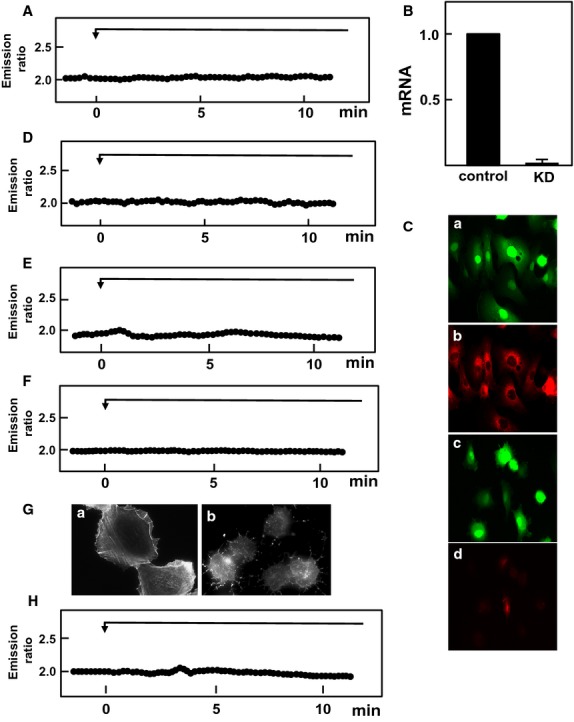
Role of TRPV2, PI 3-kinase and Rac in elevation of [Ca^2+^]_s_ induced by focal mechanical stress. (A) Mechanical stress was applied focally as shown in Figure2 and changes in [Ca^2+^]_s_ beneath the pipette were monitored in the presence of 10 μmol/L ruthenium red. The result is a representative of three experiments. (B) mRNA expression levels were measured by quantitative RT-PCR in control and TRPV2 knocked down (KD) cells. Values are the mean ± SE for four experiments. (C) Efficiency of Knockdown of TRPV2. Cells were transfected with control vector (a) or shTRPV2 (c). Protein expression of TRPV2 was visualized in b and d. Note that TRPV2 expression was almost completely abolished by shTRPV2 (d). (D) Mechanical stress was applied in a TRPV2 knocked down cell and changes in [Ca^2+^]_s_ were monitored. The result is a representative of four experiments. (E) Mechanical stress was applied in the presence of 100 μmol/L LY29034. The result is a representative of five experiments. (F) Mechanical stress was applied in cell expressing dominant negative Rac. The result is a representative of three experiments. (G) Cells were treated with or without latrunculin and actin filaments were visualized. (H) Mechanical stressed was applied in cells treated with latrunculin. The result is a representative of three experiments.

**Figure 4:**
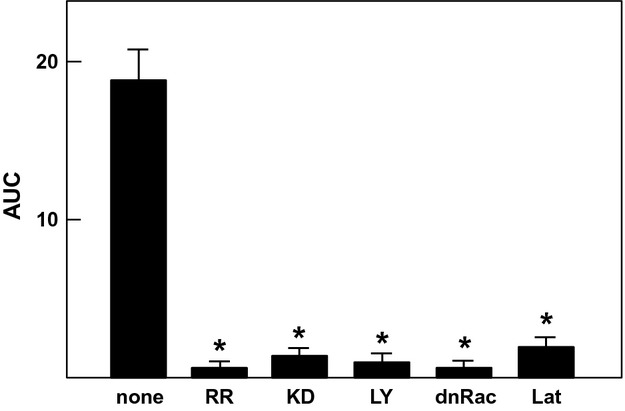
Effect of inhibition of TRPV2 on mechanical stress-induced elevation of [Ca^2+^]_s_. Experiments were carried out as shown in Fig.2B and 3. The AUC of the [Ca^2+^]_s_ response was calculated. Values are the mean ± SE for three to five experiments. * *P *<* *0.01 versus none. RR, ruthenium red; KD, knockdown of TRPV2; LY, LY29034; dnRac, dominant-negative Rac; Lat, latrunculin.

In this study, we used HT1080 cells, a cell line derived from human fibrosarcoma. We determined the expression of channels belonging to the TRPV family in these cells. As shown in Figure1A, among six members of the TRPV family, only the expression of TRPV2 was detected by RT-PCR. TRPV2 is the only member of the TRPV2 channel family expressed in these cells. Note that primers used in this study detected TRPV1 to TRPV6 in mRNA obtained from human brain and kidney (data not shown). We applied mechanical stress by attaching a glass pipette to the cell and simultaneously applying negative pressure (10 mm H_2_O). Note that the mechanical stress applied by this method was reversible and the cell shape returned normal immediately after detachment of the pipette (Fig.1B). To confirm that focal mechanical stress was in fact administered, we measured the changes in the distribution of zyxin, an actin-regulated protein known to be accumulated in the site receiving a mechanical stress (Hirata et al. 2008). As shown in Figure1C, accumulation of zyxin was observed after the application of negative pressure through the attached pipette. We then measured changes in subplasma membrane free calcium concentration ([Ca^2+^]_s_) in cells receiving focal mechanical stress. To this end, we used Yellow Cameleon-nano, a sensitive calcium indicator (Horikawa et al. 2010), targeted to the plasma membrane (pm-Cameleon-nano). We monitored changes in [Ca^2+^]_s_ using pm-Cameleon-nano. As shown in Figure1D, application of focal mechanical stress induced an immediate elevation of [Ca^2+^]_s_ beneath the pipette, that is, at the site receiving the mechanical stress. This elevation of [Ca^2+^]_s_ propagated throughout the cell. [Ca^2+^]_s_ then decreased and returned to nearly basal levels but [Ca^2+^]_s_ remained slightly elevated at the site receiving the mechanical stress. Several minutes later, a second elevation of [Ca^2+^]_s_ was observed, which also propagated throughout the cells. Oscillation of [Ca^2+^]_s_ was observed as long as the mechanical stress was applied. Figure2 demonstrates the time course of changes in [Ca^2+^]_s_ in different points of the cell. Elevation of [Ca^2+^]_s_ was rapid and sustained at the site receiving mechanical stress (Fig.2B). The initial elevation of [Ca^2+^]_s_ was propagated throughout the cells and the elevation was observed transiently (Fig.2C and D). When extracellular calcium was removed, elevation of [Ca^2+^]_s_ in response to mechanical stress was abolished (Fig.2E and F). The elevation of [Ca^2+^]_s_ in response to focal mechanical stress was not observed when ruthenium red, an inhibitor of TRPV channels, was administered (Fig.3A). We then knocked down TRPV2 in HT1080 cells. As shown in Figure3B, mRNA for TRPV2 was markedly reduced. Similarly, the protein expression of TRPV2 was markedly reduced by knockdown of TRPV2 (Fig.3C). In this condition, [Ca^2+^]_s_ response to the focal application of mechanical stress was abolished (Fig.3D). We previously showed that PI 3-kinase is involved in the regulation of TRPV2 (Kanzaki et al. 1999). We therefore examined the effect of a PI 3-kinase inhibitor LY29034. As shown in Figure3E, LY29034 inhibited mechanical stress-induced elevation of [Ca^2+^]_s_. We then examined the role of Rac in the regulation of TRPV2. As shown in Figure3F, transfection of the dominantly negative mutant of Rac abolished the elevation of [Ca^2+^]_s_ induced by the focal mechanical stress. We then disrupted actin filament by applying latrunculin, an inhibitor of actin assembly (Fig.3G). Latrunculin blocked [Ca^2+^]_s_ response to the mechanical stress (Fig.3H). Figure4 demonstrates quantitative analyses of the effects of inhibition of TRPV2 on mechanical stress-induced elevation of [Ca^2+^]_s_.

**Figure 5:**
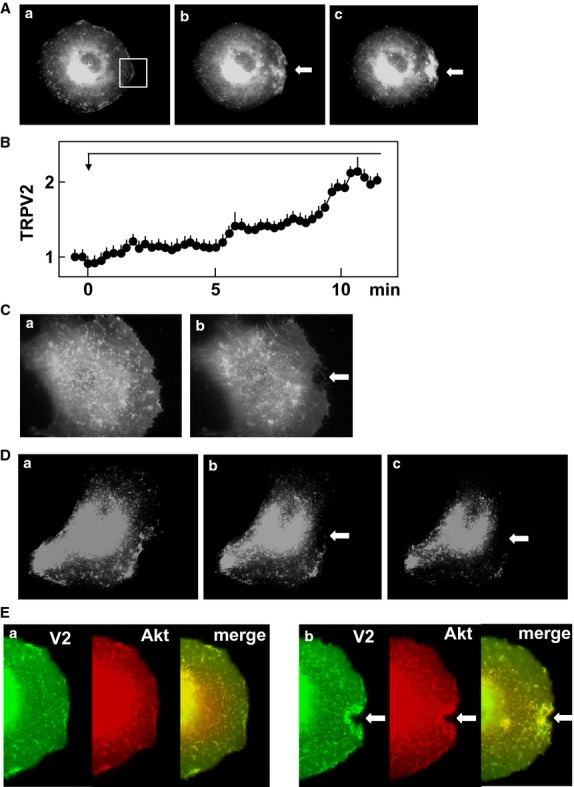
Effect of focal application of mechanical stress on localization of TRPV2. (A) Effect of focal mechanical stress on localization of TRPV2. Mechanical stress was applied at the site indicated by the arrow. Localization of GFP-TRPV2 is shown. a: before the application, b: 1 min, c: 5 min after the application. The results are the representative of more than 50 experiments. (B) Time course of accumulation of TRPV2 beneath the pipette. Mechanical stress was applied to the cells as shown in A. Changes in the intensity of fluorescence of GFP-TRPV2 in area shown in Fig.5A-a were monitored. Values are the mean ± SE for five experiments. (C) Effect of mechanical stress in the absence of extracellular calcium. Mechanical stress was applied as shown in A in the absence of extracellular calcium. The results are the representative of two experiments. a: before application of mechanical stress, b: 5 min after the application of mechanical stress. (D) Effect of LY29034 on translocation of TRPV2. Focal mechanical stress was applied as indicated above in the presence of 100 μmol/L LY29034. The results are the representative of five experiments. a: before the application, b: 1 min, c: 5 min after the application. (E) Effect focal application of mechanical stress on localization of TRPV2 and Akt. Focal mechanical stress was applied as indicated above and localization of GFP-TRPV2 (green) and RFP-Akt (red) was monitored. The results are the representative of four experiments. a: before the application, b: 5 min after the application.

We then addressed whether or not focal application of mechanical stress induced translocation of TRPV2. We transfected TRPV2-GFP and monitored changes in distribution of the GFP fluorescence. As shown in Figure5A, TRPV2-GFP was accumulated beneath the pipette. Accumulation was observed as long as the mechanical stress was applied. Figure5B shows time course of accumulation of TRPV2 beneath the pipette. As depicted, TRPV2 accumulated beneath the pipette gradually and accumulation reached the peak value around 10 min after the addition of the mechanical stress. Note that typical accumulation of TRPV2 was observed in more than 80% of the applications of the mechanical stress. After detaching the pipette, accumulation of TRPV2-GFP reduced gradually (data not shown). In the absence of extracellular calcium, mechanical stress did not cause accumulation of TRPV2 (Fig.5C).

When PI 3-kinase was inhibited, TRPV2 was not accumulated beneath the pipette even in the presence of mechanical stress (Fig.5D). We then investigated whether or not PI 3-kinase was activated locally by mechanical stress. To this end, we monitored distribution of Akt, a protein which binds to PI 3, 4, 5-trisphostate. As shown in Figure5E, both TRPV2-GFP and RFP-Akt were accumulated beneath the pipette, indicating that PI 3-kinase was activated locally by the mechanical stress and phosphorylated Akt was bound to phosphatidylinositol 3, 4, 5-trisphosphate in the membrane.

**Figure 6:**
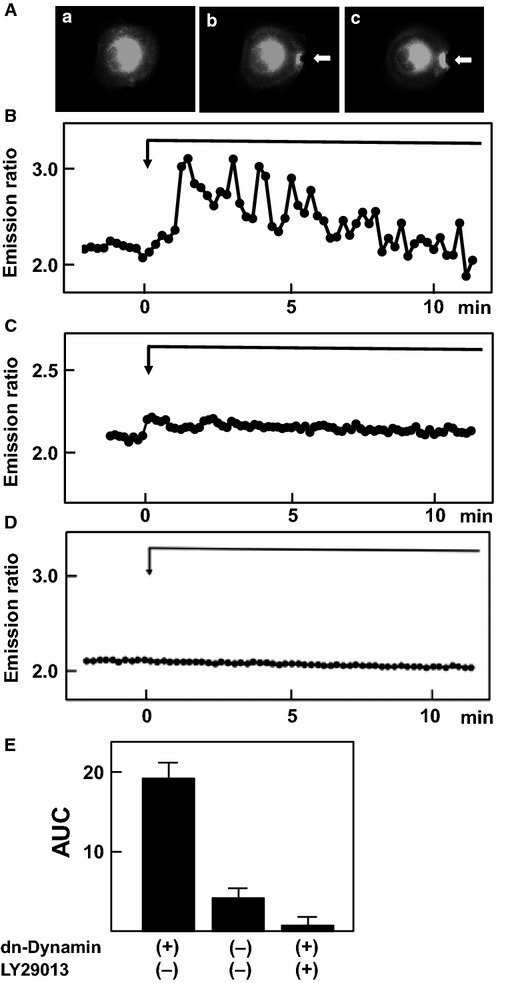
Effect of dominant-negative dynamin and LY29013. (A) Effect of dominant-negative dynamin on localization of TRPV2. Focal mechanical stress was applied to a dominant-negative dynamin-expressing cell and changes in localization of GFP-TRPV2 were monitored. The results are the representative of four experiments. a: before the application, b: 5 min after the application, c: 10 min after the removal of mechanical stress. (B) Effect of focal application of mechanical stress on [Ca^2+^]_s_ in the presence of dominant-negative dynamin. Focal mechanical stress was applied in a dominant-negative dynamin-expressing cell and changes in [Ca^2+^]_s_ beneath the pipette were monitored. The result is a representative of five experiments. (C) Effect of focal application of mechanical stress on [Ca^2+^]_s_ in the absence of dominant-negative dynamin. Experiments were carried out as in B in cells not transfected with dominant-negative dynamin. (D) Effect of LY29013 on changes in [Ca^2+^]_s_. Focal mechanical stress was applied to the dominant-negative dynamin-expressing cell in the presence of 100 μmol/L LY29013 and changes in [Ca^2+^]_s_ beneath the pipette were monitored. The results are the representative of five experiments. (E) Quantitative analysis of the effect of dominant-negative dynamin and LY29013 on [Ca^2+^]_s_. Experiments were carried out as described in B, C and D. The AUC was calculated. Values are the mean ± SE for five experiments. *P <* *0.001 versus dominant-negative dynamin-expressing cells without LY29013.

The above results indicate that focal application of mechanical stress accumulated TRPV2 to the site receiving the mechanical stress. The amount of TRPV2 in the plasma membrane is regulated in a complex manner by exocytosis and endocytosis of TRPV2 (Kojima and Nagasawa 2007). To further study the regulation of TRPV2, we blocked both exocytosis and endocytosis of TRPV2 and the effect of mechanical stress was observed. To block endocytosis, we used a dominant-negative mutant of dynamin (Omata et al. 1997). As shown in Figure6A, when a cell expressing dominant-negative dynamin received focally applied mechanical stress, TRPV2-GFP was accumulated considerably beneath the pipette. TRPV2-GFP remained accumulated even after the mechanical stress was removed, an observation compatible with the notion that endocytosis of TRPV2 was inhibited. Under the same condition, application of the focal mechanical stress induced a marked increase in [Ca^2+^]_s_ as shown in Figure6B. Figure6C shows time course of changes in [Ca^2+^]_c_ in cells not expressing dominant-negative dynamin. As depicted, oscillation of [Ca^2+^]_s_ was marked and the amplitude of [Ca^2+^]_s_ responses was much greater in cells expressing dominant-negative dynamin. When LY29042, an inhibitor of PI 3-kinase, was administered to the cell expressing dominant-negative dynamin, focally applied mechanical stress did not increase [Ca^2+^]_s_ (Fig.6D). Figure6E depicts quantification of the results. Similar results were obtained when the cell was transfected with dominant-negative dynamin and Rac instead of adding LY29042 (data not shown).

**Figure 7:**
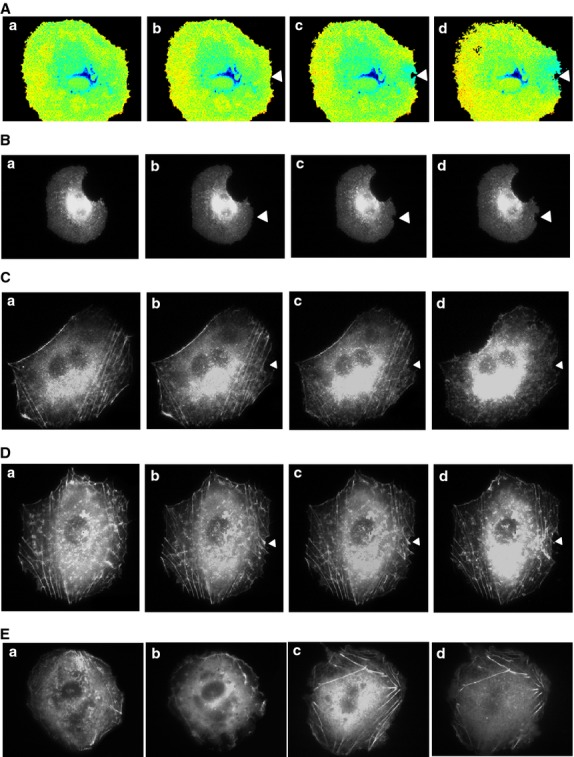
Effect of Gadolinium on TRPV2. (A) Effect of Gadolinium on [Ca^2+^]_s_. Focal mechanical stress was applied to the site indicated by the arrow head in the presence of 10 μmol/L gadolinium and changes in the [Ca^2+^]_s_ were monitored using pm-Cameleon-nano. The results are the representative of four experiments. a: before the application, b: 1 min, c: 3 min, d: 5 min after the application. (B) Effect of Gadolinium on the localization of TRPV2. Focal mechanical stress was applied as indicated by the arrow in the presence of 10 μmol/L gadolinium. Changes in the localization of GFP-TRPV2 were monitored. The results are the representative of four experiments. a: before the application, b: 1 min, c: 3 min, d: 5 min after the application. (C) Effect of focal application of mechanical stress on actin filament. Focal mechanical stress was applied as indicated by the arrowhead and changes in actin filaments were monitored. The results are the representative of four experiments. a: before the application, b: 3 min, c: 5 min, d: 10 min after the application. (D) Effect of Gadolinium of actin filaments. Focal mechanical stress was applied in the presence of 10 μmol/L gadolinium and changes in the actin filaments were monitored. The results are the representative of four experiments. a: before the application, b: 3 min, c: 5 min, d: 10 min after the application. (E) Effect of Gadolinium on EGF-induced changes in actin filaments in the absence of extracellular calcium. Cells were incubated in medium containing no calcium and stimulated with 10 nmol/L EGF (b, d) in the presence (c, d) and absence (a, b) of 10 μmol/L gadolinium and actin filaments were monitored. The results are the representative of two experiments. a, c: before stimulation, b, d: 10 min after the addition of EGF.

It is well known that gadolinium is an inhibitor of mechanosensitive channels (Hamill and McBride 1996). As shown in Figure7A, administration of gadolinium completely blocked the elevation of [Ca^2+^]_s_ induced by focally applied mechanical stress. We then examined whether or not gadolinium affected the accumulation of TRPV2 beneath the pipette. As depicted in Figure7B, accumulation of TRPV2-GFP beneath the pipette was blocked in the presence of gadolinium. Thus, gadolinium nearly completely blocked accumulation of TRPV2-GFP to the site receiving the mechanical stress. To determine the effect of gadolinium on cytosketal structures, we monitored changes in the actin filaments in response to focally applied mechanical stress. As shown in Figure7C, application of mechanical stress disrupted stress fibers around the pipette. In contrast, disruption of actin filaments was not induced by the mechanical stress in the presence of gadolinium (Fig.7D). To determine if gadolinium has some direct effects on cytoskeleton, we added gadolinium to cells stimulated by epidermal growth factor (EGF) in the absence of extracellular calcium. As shown in Figure7E, gadolinium affected the changes in actin filaments induced by EGF in the absence of extracellular calcium.

**Figure 8:**
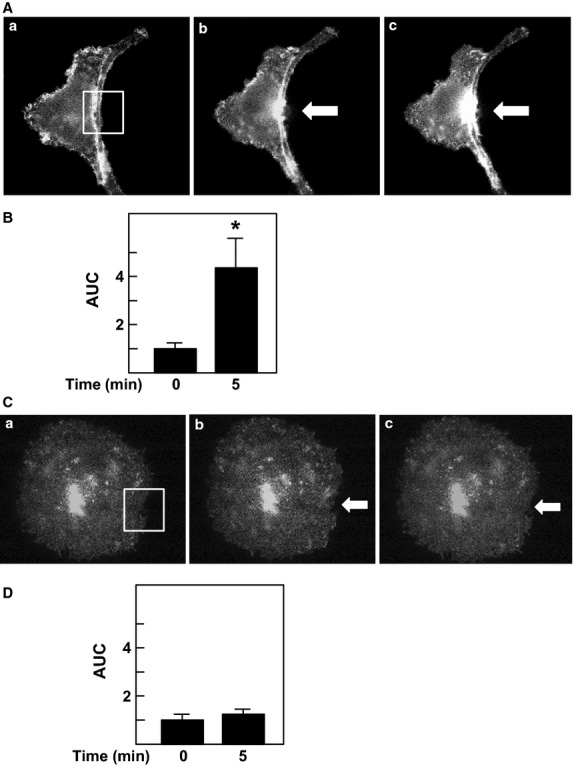
Effect of focal application of mechanical stress on localization of TRPV4 and TRPV1. (A) Effect of focal mechanical stress on localization of TRPV4. Mechanical stress was applied at the site indicated by the arrow. Localization of GFP-TRPV4 is shown. The results are the representative of ten experiments. a: before the application, b: 1 min after the application, c: 5 min after the application. (B) Quantification of the effect of mechanical stress. Experiments were carried out as shown in A. Changes in fluorescence intensity of GFP-TRPV4 in area shown in A-a were monitored and AUC was calculated. Values are the mean ± SE for ten experiments. *P <* *0.05 versus time 0. (C) Effect of Focal Mechanical Stress on Localization of TRPV1. Mechanical stress was applied at the site indicated by the arrow. Localization of GFP-TRPV1 is shown. The results are the representative of 10 experiments. a: before the application, b: 1 min after the application, c: 5 min after the application. (D) Quantification of the effect of mechanical stress. Experiments were carried out as shown in C. Changes in fluorescence intensity in area shown in C-a were monitored and AUC was calculated. Values are the mean ± SE for ten experiments.

The above results show that TRPV2 translocates to the site receiving the mechanical stress in HT1080 cells. Using this system, we next addressed whether or not other members of the TRPV family have a similar function. We ectopically expressed GFP-tagged TRPV1, TRPV3, TRPV4 TRPV5, or TRPV6 in HT1080 cells, and examined whether these channels were accumulated to the site receiving mechanical stress. As shown in Figure8A and B, TRPV4-GFP accumulated beneath the pipette, indicating that TRPV4 translocated to the plasma membrane in response to the mechanical stress. In the presence of LY29034, translocation of TRPV4 was not observed. In contrast, TRPV1-GFP did not accumulate beneath the pipette (Fig.8C and D). Other members of the TRPV family including TRPV3-GFP, TRPV5-GFP, and TRPV6-GFP did not accumulate in response to the mechanical stress (data not shown).

## Discussion

In this study, we investigated the modulation of TRPV2 by focal administration of mechanical stress. We administered reversible focal mechanical stress by applying negative pressure using a glass pipette (Fig.1B). As shown in Figure1C, focal application of the mechanical stress induced accumulation of zyxin to the area beneath the pipette. As zyxin accumulates when a mechanical stress is administered (Hirata et al. 2008), the result shown in Figure1C confirmed that focal mechanical stress was in fact applied by our method. As shown in Figure1D, focal mechanical stress induced a rapid elevation of [Ca^2+^]_s_, which propagated throughout the cell. These oscillatory changes in [Ca^2+^]_s_ were totally dependent on extracellular calcium and were blocked by ruthenium red and also by knockdown of TRPV2. Hence, focal application of mechanical stress induces elevation of [Ca^2+^]_s_ by a mechanism involving TRPV2. Then the question arises as to whether or not the mechanical stress activates TRPV2 directly by modifying the gating of the channel or by other mechanisms, for example, by accumulating TRPV2 at the site receiving the mechanism stress. We therefore determined changes in localization of TRPV2 in cells expressing GFP-TRPV2. As shown in Figure5A, GFP-TRPV2 was accumulated at the site receiving the mechanical stress. The result is consistent with the notion that translocation of TRPV2 is regulated by a variety of stimuli including membrane stretch. As in cardiomyocytes (Iwata et al. 2003), TRPV2 translocates to the plasma membrane when the plasma membrane is stretched. The present results extend further in that TRPV2 accumulates to a small area receiving the focal mechanical stress. Previous studies (Kanzaki et al. 1999; Boels et al. 2001; Nagasawa et al. 2007; Hisanaga et al. 2009; Monet et al. 2009; Nagasawa and Kojima 2012) have shown that activation of PI 3-kinase is critical for translocation of TRPV2. Results shown in Figure5E indicate that Akt was also accumulated beneath the pipette. This observation suggests that PI 3, 4, 5-trisphosphate, a product of PI 3-kinase, is accumulated by the pipette. It is known that mechanical stresses applied to the cell activate PI 3-kinase (Sedding et al. 2003; Kippenberger et al. 2005), although the precise mechanism for the activation is still elucidative. The present results extend in that focal administration of mechanical stress activates PI 3-kinase locally, causes production of PI 3, 4, 5-trisphosphate and induces accumulation of Akt. This explains the effectiveness of PI 3-kinase inhibitor LY29034 in blocking the elevation of [Ca^2+^]_s_ induced by the focal application of the mechanical stress. Collectively, locally applied mechanical stress or membrane stretch activates PI 3-kinase locally and induces translocation of TRPV2 to the site receiving the mechanical stress, leading to an elevation of [Ca^2+^]_s_. Consequently, activation of TRPV2 by the mechanical stress observed in vascular smooth muscle cells (Muraki et al. 2003) and endothelial cells (Ito et al. 2010) may be due to stretch-induced translocation of TRPV2. In addition, activation of TRPV2 in neurite (Shibasaki et al. 2010) may be at least partly due to translocation of TRPV2 to the site receiving the mechanical stress. We previously reported that TRPV2 accumulates around the podosome in migrating macrophages (Nagasawa and Kojima 2012). Given that podosome is a special form of adhesion apparatus receiving a large mechanical force (Linder and Appfelbacher 2003), TRPV2 may accumulate to the podosome and possibly other types of adhesion apparatus by a similar mechanism. When the time courses of accumulation of TRPV2 (Fig.5B) and elevation of [Ca^2+^]_s_ (Fig.2B) are compared, the elevation of [Ca^2+^]_s_ is observed earlier than the accumulation of TRPV2. This suggests that the initial elevation of [Ca^2+^]_s_ is not due to translocation of TRPV2. Instead, it is presumably due to activation of TRPV2 already expressed in the plasma membrane (Pollosin et al. 1848). Collectively, focal mechanical stress elevates [Ca^2+^]_s_ by two mechanisms: direct activation of preexisting TRPV2 in the plasma membrane and subsequent recruitment of TRPV2 from intracellular compartments which is dependent on both calcium and PI 3-kinase.

To further assess the regulation of TRPV2, we blocked endocytosis of TRPV2 using dominant-negative dynamin. We previously showed that dynamin regulates endocytosis of TRPV2 (Kojima and Nagasawa 2007; Nagasawa et al. 2007). As shown in Figure6A, dominant-negative dynamin effectively fixed TRPV2 in the plasma membrane. In this condition, elevation of [Ca^2+^]_s_ induced by the focal mechanical stress was abolished when exocytosis of TRPV2 was blocked by an inhibitor of PI 3-kinase (Fig.6C). Indeed, the elevation of [Ca^2+^]_s_ was completely blocked by the inhibitor of PI 3-kinase. Accordingly, in addition to translocation of TRPV2, activation of preexisting TRPV2 in the plasma membrane was also dependent on the PI 3-kinase activity.

Gadolinium has been widely used as a blocker of the mechanosensitive channels (Sachs 1988; Hamill and McBride 1996). As shown in Fig.7A, gadolinium nearly completely blocked elevation of [Ca^2+^]_s_ by focal application of mechanical stress. Interestingly, gadolinium blocked accumulation of TRPV2 beneath the pipette. In addition to the direct inhibition of gating of the TRPV2 channel, gadolinium may also affect translocation in these cells. This is at least partly due to the inhibition of Ca^2+^ entry by gadolinium as mechanical stress-induced translocation of TRPV2 is dependent on calcium. Note that treatment with gadolinium affected the changes in stress fibers induced by EGF in the absence of extracellular calcium. It seems likely that gadolinium also modifies regulation of cytoskeletal proteins including actin filaments and indirectly regulates translocation of TRPV2.

In HT1018 cells, TRPV2 is the only member of the TRPV family channel expressed in these cells. We were able to show that exogenous GFP-TRPV2 accumulated at the site receiving mechanical stress. This experimental system enabled us to assess if the other members of the TRPV family have a similar function. We therefore examined whether or not other members of the TRPV family behaved in a similar manner as TRPV2. When expressed ectopically in HT1080 cells, other members of the TRPV family except TRPV4 were not accumulated to the site receiving mechanical stress. As shown in Fig.8A and 8B, GFP-TRPV4 behaved in a similar manner as TRPV2 and was accumulated to the area beneath the pipette. TRPV4 is thought to be regulated by osmolarity (Liedtke 2006; Liedtke and Friedman 2008) and mechanical stress (Ito et al. 2010). It is reasonable to speculate that changes in osmolarity and other mechanical stress induce translocation of TRPV4 and elevate [Ca^2+^]_s_. However, we cannot totally exclude the possibility that TRPV2 and TRPV4 form a heterodimer and translocate to the site receiving mechanical stress. In conclusion, TRPV2 and possibly TRPV4 accumulate to the site receiving mechanical stress and contribute to the increase in [Ca^2+^]_s_ in the localized area. These channels would respond to mechanical stress or membrane stretch and exert unique roles in mechanosensing.
